# Correlation of Cutaneous Immunoreactants in Lesional Skin with the Serological Disorders and Disease Activity of Systemic Lupus Erythematosus

**DOI:** 10.1371/journal.pone.0070983

**Published:** 2013-08-05

**Authors:** Yi-jin Luo, Guo-zhen Tan, Min Yu, Kai-wen Li, Yue-yang Liu, Qing Guo, Fan-qin Zeng, Liangchun Wang

**Affiliations:** 1 Department of Dermatology, Sun Yat-sen Memorial Hospital, Sun Yat-sen University, Guangzhou China; 2 Department of Dermatology, The 7th Hospital of Shenyang, Shenyang, China; Wayne State University, United States of America

## Abstract

Detection of immunoreactants including IgG, IgM, IgA, and C3 by direct immunofluorescence (DIF) from skin is useful for distinguishing lupus lesions from other skin disorders. Despite their diagnostic value, the type and number of cutaneous immunoreactants as they relate to serological disorders and disease severity has been poorly studied. We examined 36 patients with systemic lupus erythematosis (SLE) with positive DIF (DIF+) and 28 patients with negative DIF (DIF−) tests performed on lesional skin. Among DIF+ patients, the most frequent patterns of immunoreactants were IgM alone (36%) and the coexistence of IgM with C3 (28%). IgM was the highest detected individual immunoreactant (86%). As classified by number, 17 of 36 DIF+ patients had one immunoreactant ( = 1), while the remaining patients had two to four immunoreactants (>1). Compared with DIF− patients, DIF+ patients were more likely to have severe disease as indicated by lower serum C3 levels and a higher SLE disease activity index (SLEDAI). The coexistence of IgM with any other immunoreactants indicated a more severe disease than that present in the DIF− group, whereas the IgM-alone group was comparable with the DIF− group in both serum C3 levels and SLEDAI. These findings were also applicable in the comparison of patients with more than one (>1) immunoreactant and patients with no (DIF−) and one ( = 1) immunoreactant. Collectively, the presence of multiple immunoreactants in lesional skin implies a more severe disease activity of SLE, while a single immunoreactant may be equal to the absence of immunoreactants (DIF−) in terms of predicting disease activity.

## Introduction

Systemic lupus erythematosus (SLE) is a systemic autoimmune disease with frequent involvement of the skin. Clinically, the presence of a skin rash is important as it is one of the earliest symptoms that patients report [1]. Diagnosis of lupus lesions in patients with skin rashes is determined by a clinical test using direct immunofluorescence (DIF) to detect immunoreactants, most commonly immunoglobulin G (IgG), IgM, IgA, and complement component 3 (C3), along the dermal-epidermal junction [2]–[4].

Despite their diagnostic value, tests to determine the presence of cutaneous immunoreactants in lupus lesions have not been used to study disease progression with other organ injuries and serological disorders characteristic of SLE. However, the presence of these immunoreactants in nonlesional skin has been suggested to indicate a lower 10-year survival rate [5] and lower serum levels of C3 [6], [7]. In addition, the presence of multiple immunoreactants in lesions reportedly indicates more active disease as measured by the SLE disease activity index (SLEDAI) [8]–[10]. However, this concept has been challenged by other studies [9], [11].

Due to the fact that most DIF tests are performed during the early stage of skin lesions, few studies on immunoreactants in lesional skin have been performed. Our previous research showed that the detection rate of immunoreactants in lesional skin varied from 30% to 50% and that IgM was the most frequent immunoreactant [12], which is consistent with other published data [3], [13]–[15]. We enrolled 64 patients diagnosed with SLE and examined DIF conducted on lesional skin to assess whether the type and number of cutaneous immunoreactants present in the lesional skin correlated with serological disorders and disease severity as measured by the SLEDAI.

## Materials and Methods

### Ethics Statement

The analysis was conducted on anonymized data that had been collected as part of routine patient care. No additional investigations were performed. Therefore, no prior informed consent from the patients was required. For clinical pictures, the individual has given written informed consent, as outlined in the PLOS consent form, to publication of their photograph. The study was carried out in accordance with the Declaration of Helsinki and was approved by the research ethics board of Sun Yat-sen Memorial Hospital. Our ethics committee waived the need for informed consent.

### Patients

All patients were diagnosed with SLE according to the 1997 American College of Rheumatology Revised Criteria for Classification of SLE [16]. Disease activity was measured with the SLEDAI. Eligible laboratory parameters were those collected around the time that the skin biopsy was performed and included a complete blood count, erythrocyte sedimentation rate (ESR), and levels of serum C3, anti-nuclear antibody (ANA), anti-dsDNA antibody, and extractable nuclear antibodies (anti-SSA, SSB, RNP, and Sm antibodies).

### Direct Immunofluorescence

All DIF examinations were performed on lesional skin. Briefly, fresh skin samples were embedded in OCT tissue-freezing medium and cut into sections with a thickness of 0.5 µm in a cryostat. For staining, sections were brought to room temperature, washed twice with phosphate-buffered saline (PBS), and incubated with fluorescein isothiocyanate-conjugated rabbit anti-human IgG, IgA, IgM, and C3 antibodies in a humidified chamber for 30 minutes at room temperature. Unbound antibodies were washed off with PBS. The sections were viewed under an ultraviolet microscope.

### Statistical Analysis

Pearson’s chi-square test was used for all enumeration data. Comparison of measurement data was conducted with the Mann-Whitney test for two groups and with one-way analysis of variance followed by Dunn’s post hoc test for three groups.

## Results

### IgM alone and the Combination of IgM+C3 were the Most Frequent Patterns Detected from Lesional Skin

**Figure 1:**
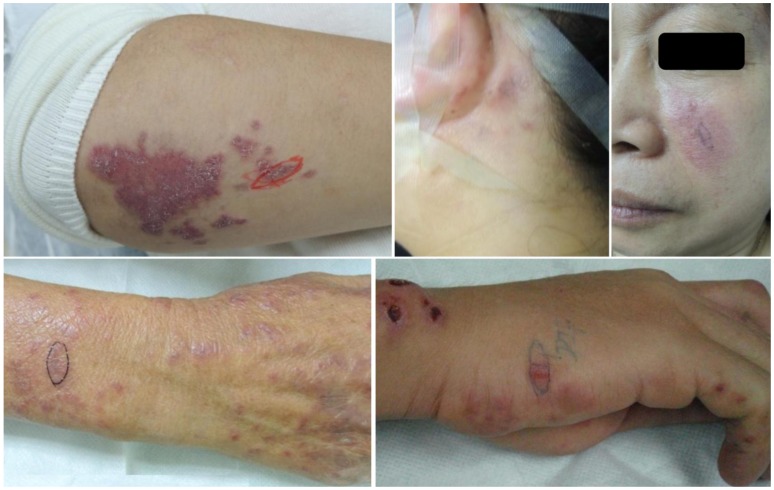
Cutaneous lesions in SLE patients. The skin injury of SLE presents multiple morphologies, including atrophic scaly purplish-red macules, papules and plaques, indurate erythema and vacuities as well.

**Figure 2:**
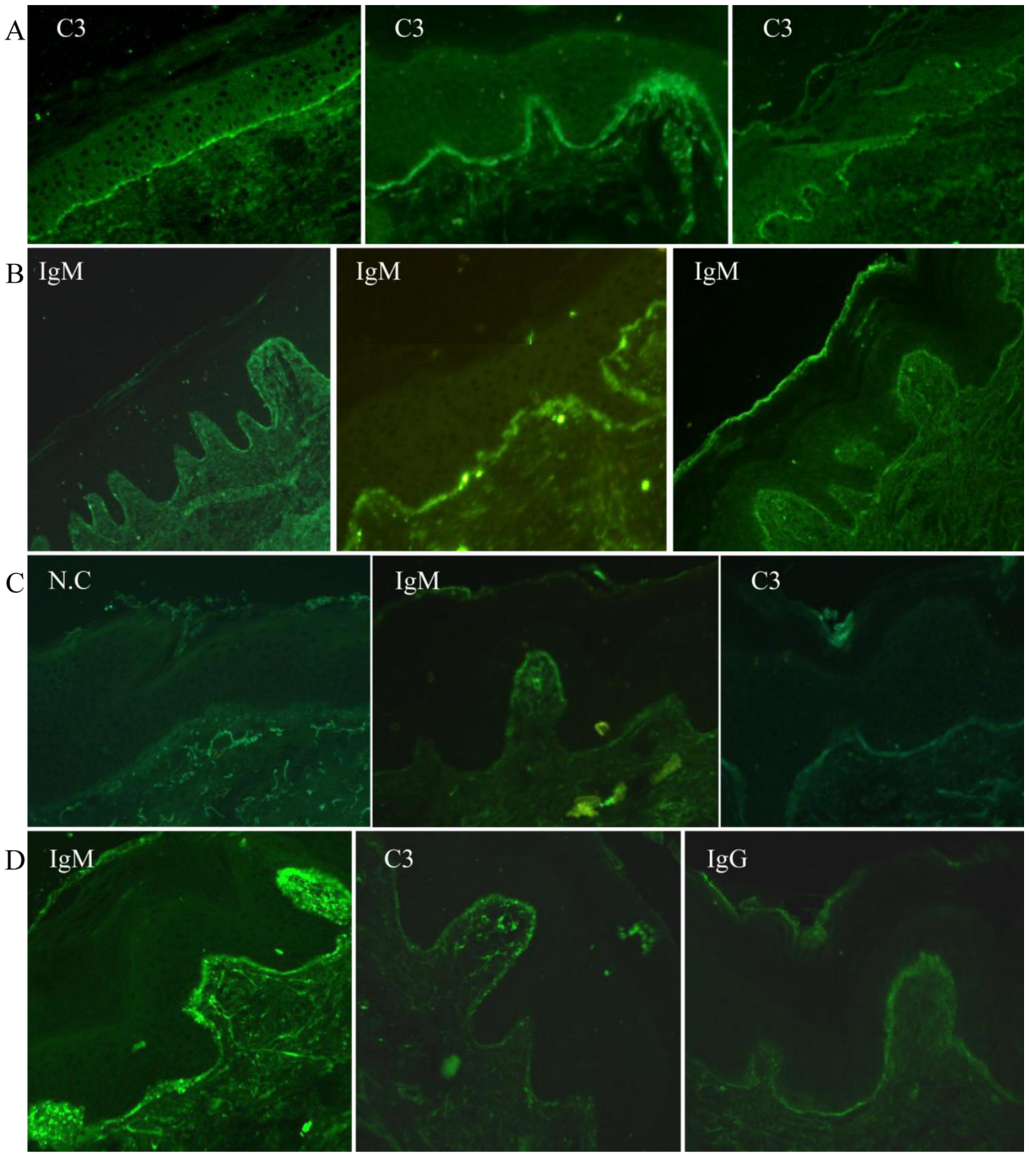
Deposit of immunoreactants along epidermal-dermal junction in lesional skin. Skin sections were incubated with fluorescein isothiocyanate (FITC)-conjugated anti-human IgG, IgA, IgM, and C3 antibodies and visualized with a fluorescence microscope. A and B indicates the staining patterns of single immunoreactant detected from individual biopsy. The representative staining of the coexistence of two and three immunoreactants from two biopsies were shown in C and D, respectively. N.C, negative control without adding FITC-conjugated antibody.

Sixty-four patients were involved in this study. There were 48 females and 16 males (F:M = 3∶1) with average ages of 33±15 and 31±16 years, respectively. The disease duration, from the first appearance of SLE-related disorders to the time of performing skin biopsy, ranged from 0.25 to 132 months (19.7±33.2, mean±SD). Most of the skin lesions involved face, V-area of the neck, upper back and extensor aspects of the arms, and less involved abdomen, lower back and lower extremities. Lesions were observed localized or symmetrically generalized. The manifestations varied among sharply demarcated erythematous macules, papules, atrophic scaly purplish-red macules, indurate nodules or plaques and even cutaneous vasculitis (Figure 1). Of 64 patient samples, immunoreactants were detected along the dermal-epidermal junction in lesional skin in 36 samples (56.3%). Figure 2 showed the single, double and triple staining patterns of imunoreactants. All these patterns were present in a common continuous pattern. The brightness varied from moderate to marked. The fluorescence were seen as homogenous or solid well-demarcated band consisting of multiple small round bright points or clumps (Figure 2).The most frequently detected patterns of immunoreactants were IgM alone (13, 36%), IgM and C3 (10, 28%), and IgM/G/A and C3 (5, 14%). The frequency of each immunoreactant was 86% (IgM), 56% (C3), 25% (IgG), and 22% (IgA) (Table 1).

**Table 1:** Patterns of immunoreactants in the lesional skin of patients with SLE.

	C3	IgM	IgM C3	IgA, G, M, C3	IgG, M, C3	IgA, G, M	IgA, G	In total (36)			
								IgA	IgG	IgM	C3
Patients, n (%)	4 (11)	13 (36)	10 (28)	5 (14)	1 (3)	2 (5)	1 (3)	8 (22)	9 (25)	31 (86)	20 (20)

### Skin Lesions with IgM and Other Immunoreactants, but not IgM alone, Correlated with a Lower Serum C3 Level and more Severe Disease

First, we determined the association between the presence of cutaneous immunoreactants and serological disorders. Patients were simply divided into DIF+ and DIF− groups. The detection rates of SLE-related antibodies including ANA, dsDNA, SSA, SSB, RNP, and Sm were comparable between the two groups. The serum level of C3 in DIF+ patients was 448±197 mg/L (mean ± SD), lower than that in DIF− patients (580±232 mg/L, P<0.05), indicating a severe form of SLE in DIF+ patients. In agreement with the serum C3 result, DIF+ patients demonstrated a higher disease activity according to the SLEDAI compared with DIF− patients (Table 2). We also performed a complete blood cell count and determined the ESR, both of which are routine laboratory parameters that reflect SLE disease activity. There were no statistically significant differences between the DIF+ and DIF− groups in terms of the proportions of patients with blood cell counts below the normal ranges, the degree of decrease in each blood cell lineage, or the ESR (data not shown).

**Table 2:** Association of cutaneous IgM with serological disorders and SLEDAI.

Groups (n)		Serological disorders							SLEDAI*
		ANA	dsDNA	SSA	SSB	RNP	Sm	Serum C3§	Mean
DIF− (28)		22	18	11	2	5	4	580±232	6.0±3.0
DIF+	DIF+(36)	34	24	13	11	12	9	448±197	8.9±4.6
	IgM alone(13)	11	9	5	4	5	3	538±222	6.8±3.9
	IgM+ other(18)	18	13	5	0	6	5	359±148	9.6±3.7

§ P<0.05: DIF+ versus DIF−, IgM+other versus DIF−.

* P<0.05: DIF+ versus DIF−, IgM+other versus DIF−.

**Figure 3:**
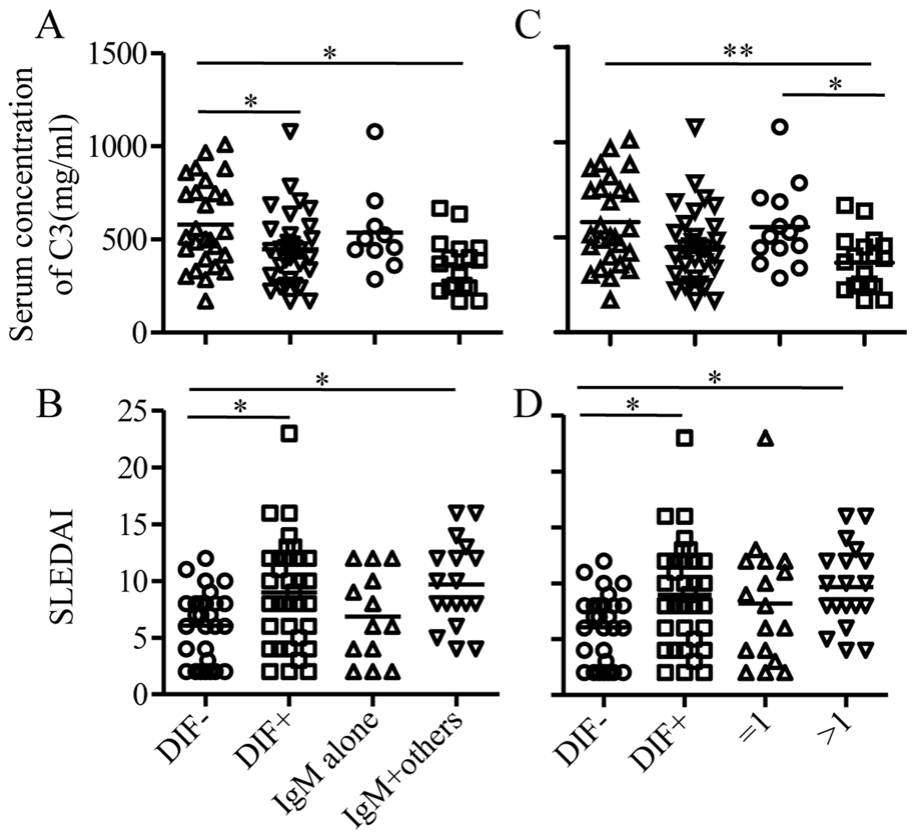
The comparison of serum C3 concentration and SLEDAI in groups of patients Patients were first divided into DIF− and DIF+ groups. DIF+ group was then divided into two subgroups according to the existence pattern (A, B) and the number of immunoreactants(C, D), respectively. Serum C3(A, C) and SLEDAI(B,D) were compared among each of four groups. Each symbol represents one individual, and the bar indicates the mean. Statistical analysis was performed with one-way analysis of variance followed by Dunn’s post hoc test for three groups. *P<0.05, ** *P*<0.01.

Second, because IgM was the most frequent immunoreactant, we determined whether cutaneous IgM deposits are associated with serological disorders. DIF+ patients were divided into three subgroups according to the pattern of cutaneous IgM (number of patients): IgM alone (13), IgM+ other immunoreactant (18), and IgM negative and DIF positive (IgM-DIF+) (5). The subsequent analysis focused on the first two subgroups because only five patients were IgM- DIF+. The presence of SLE-related antibodies including ANA, dsDNA, SSA, RNP, and Sm did not differ among the IgM-alone, IgM+other immunoreactants, and DIF− groups (P>0.05). Interestingly, none of the 18 patients with IgM+other immunoreactants in the skin showed SSB-positive serum samples, whereas 7 SSB-positive serum samples were detected from the other groups (2 of 28 DIF− patients, 1 of 5 IgM-DIF+ patients, and 4 of 13 IgM alone patients) (Table 2). The serum level of C3 in the group of patients with cutaneous IgM+other immunoreactants (359±148, mean ± SD) was lower than that in the DIF− group (580±232), but comparable with that in the IgM-alone group (538±222), whereas there were no statistically significant differences between the latter two groups (Figure 3A, Table 2). These results suggest that the pattern of cutaneous IgM along with other immunoreactants is related to a higher disease activity. This was further confirmed by SLEDAI analysis. The SLEDAI of 18 patients with cutaneous IgM+other immunoreactants was 9.6±3.7 (mean ± SD), higher than that in the DIF− group (6.8±3.9) but comparable with the IgM-alone group (6.8±3.9); the latter two groups showed no statistical significance (P>0.05) (Figure 3B, Table 2). Comparisons among these groups were also performed in terms of the complete blood cell count and ESR, and revealed no association with the pattern of cutaneous IgM (data not shown).

### Number of Cutaneous Immunoreactants Correlated with Serological Disorders and Disease Severity

We concluded that in lesional skin, the presence of IgM along with other immunoreactants, but not IgM alone, correlated with a lower serum C3 level and higher SLEDAI. Previous studies conducted in nonlesional skin demonstrated that the detection of multiple rather than single immunoreactants likely implied a higher disease activity [8]. Accordingly, we tested whether the current findings are a unique feature of cutaneous IgM or a common phenomenon also applicable to the other immunoreactants in lesional skin. DIF+ patients were divided into 2 groups according to the number of cutaneous immunoreactants: 17 patients with a single immunoreactant ( = 1) and 19 patients with more than 1 immunoreactant (>1). Serum ANA, dsDNA, SSA, RNP, and Sm antibody levels were comparable between the two groups and with the DIF− group. The serum C3 level was lower in the group with more than one immunoreactant (>1) than in the group with one immunoreactant ( = 1) (P<0.05), and even lower than that in the DIF− group (P<0.01), whereas the latter two groups showed no difference. SLEDAI in the group with immunoreactants (>1) was greater than that in the other two groups (Figure 3C,D; Table 3). DIF− patients and patients with one immunoreactant ( = 1) showed no statistically significant differences in either the serum C3 level or SLEDAI.

**Table 3:** Association of the number of cutaneous immunoreactants with serological disorders and SLEDAI.

Groups (n)	Number of IR (n)	Serological disorders							SLEDAI*
		ANA	dsDNA	SSA	SSB	RNP	Sm	Serum C3§	Mean
DIF+(36)	= 1 (17)	15	10	8	5	6	4	553±208	8±5
	>1 (19)	19	14	5	0	6	5	366±147	10±4

Abbreviations: IR, immunoreactant.

§ P<0.05: Immunoreatants (>1) versus immunoreactant ( = 1); P<0.01: Immunoreatants (>1) versus DIF−.

* P<0.05: Immunoreactant (>1) versus DIF−.

## Discussion

In the present study, we found that the staining pattern of immunoreactants in SLE skin lesions was commonlyobserved as a homogeneous, granular or solid well-defined fluorescent band along the dermal-epidermal junction. IgM was the most frequent immunoglobulin detected from lesional skin in patients with SLE; (2) DIF+ patients were likely to have more severe disease as indicated by a lower serum C3 level and higher SLEDAI compared with DIF− patients; (3) the presence of IgM with any other immunoreactant indicated more severe disease than that in the DIF− groups, whereas the IgM alone group was comparable with the DIF− groups in both serum C3 level and SLEDAI; and (4) regardless the type of immunoreactants, patients with more than one immunoreactant (>1) tended to have much more severe disease than those with no immunoreactants (DIF−).

The staining pattern of immunoreactants in lesional and non-lesional skin of lupus patients were thoroughly discussed by Reich A et al [4]. In the present study, the common feature was observed as a continuous fluorescent band with moderate to marked intensity consisting multiple bright dots or clumps, consistent with previous report [4]. For single immunoreactant deposition, the staining pattern varied among individual samples. For double and triple immunoreactants deposition, the staining pattern of each immunoreactant was different within the same sample. As shown in figure 2D, IgM was seen as a thicker, brighter band with clumps, C3 as a thinner band with several bright points and less intensity, while IgG as a homogenous band even though all sections were from one sample. Therefore, the staining pattern may not be associated with manifestations of skin lesions as previously discussed [4]. Our study indicates that although the pattern of immunoreactants is not indicative of disease progression, the specific immunoreactants present in the skin lesion can correlate with disease severity and progression.

These results are consistent with those of other studies showing IgM in nonlesional skin of patients with SLE [8], [17]; these studies also demonstrated that the presence of IgM alone had no correlation with clinical parameters, but the presence of IgM with either IgG, IgA, or C3 indicated a more severe form of disease. To interpret these data, first, future studies must include more patients with DIF+IgM- to predict the role of IgM in the development of cutaneous injury. Second, the present data, together with those of previous studies, are unable to rule out the possibility that all findings were unique to IgM or to claim that the number rather than the type of immunoreactants was a more predictable factor in evaluating SLE activity because the group of patients with one immunoreactant mainly comprised IgM-alone patients. In our study, 13 of 17 (76.5%) patients with one immunoreactant were IgM-positive, and the remaining 4 were C3-positive. In a previous report that showed findings identical to ours but that involved nonlesional skin [8], 22 of 26 (84.6%) patients with a single immunoreactant were IgM-positive. Thus, more patients with types of solitary cutaneous immunoreactant deposits other than IgM are required to determine whether the number or type of immunoreactants are more sensitive in predicting disease severity and activity. This may take a long time or require multiple centers working together to collect sufficient data because IgM is the dominant immunoreactant.

Taken together, the presence of multiple immunoreactants in lesional skin correlates with a more severe disease activity of SLE, while single immunoreactants may be equal to the absence of immunoreactants (DIF−) in predicting disease activity.
